# UV Protective, Antioxidant, Antibacterial and Compostable Polylactic Acid Composites Containing Pristine and Chemically Modified Lignin Nanoparticles

**DOI:** 10.3390/molecules26010126

**Published:** 2020-12-29

**Authors:** Ema Cavallo, Xiaoyan He, Francesca Luzi, Franco Dominici, Patricia Cerrutti, Celina Bernal, Maria Laura Foresti, Luigi Torre, Debora Puglia

**Affiliations:** 1 Grupo de Biotecnología y Materiales Biobasados, Facultad de Ingeniería, Instituto de Tecnología en Polímeros y Nanotecnología (ITPN-UBA-CONICET), Universidad de Buenos Aires, C1127AAR Buenos Aires, Argentina; eccavallo@fi.uba.ar (E.C.); pcerrutti@fi.uba.ar (P.C.); mforesti@fi.uba.ar (M.L.F.); 2 Facultad de Ingeniería, Universidad de Buenos Aires, C1127AAR Buenos Aires, Argentina; cbernal@fi.uba.ar; 3 Consejo Nacional de Investigaciones Científicas y Técnicas (CONICET), C1127AAR Buenos Aires, Argentina; 4 Civil and Environmental Engineering Department, University of Perugia, UdR INSTM, 05100 Terni, Italy; pghxy@hotmail.com (X.H.); francesca.luzi@unipg.it (F.L.); franco.dominici@unipg.it (F.D.); luigi.torre@unipg.it (L.T.); 5 Department of Materials Technology and Engineering, Research Institute of Zhejiang University, Taizhou 317000, China; 6 Grupo de Ingeniería en Polímeros y MAteriales Compuestos, Facultad de Ingeniería, Instituto de Tecnología en Polímeros y Nanotecnología (ITPN-UBA-CONICET), Universidad de Buenos Aires, C1127AAR Buenos Aires, Argentina

**Keywords:** polylactic acid, nanocomposite films, lignin nanoparticles, chemical modification, food packaging

## Abstract

Polylactic acid (PLA) films containing 1 wt % and 3 wt % of lignin nanoparticles (pristine (LNP), chemically modified with citric acid (caLNP) and acetylated (aLNP)) were prepared by extrusion and characterized in terms of their overall performance as food packaging materials. Morphological, mechanical, thermal, UV–Vis barrier, antioxidant and antibacterial properties were assayed; appropriate migration values in food simulants and disintegration in simulated composting conditions were also verified. The results obtained indicated that all lignin nanoparticles succeeded in conferring UV-blocking, antioxidant and antibacterial properties to the PLA films, especially at the higher filler loadings assayed. Chemical modification of the fillers partially reduced the UV protection and the antioxidant properties of the resulting composites, but it induced better nanoparticles dispersion, reduced aggregates size, enhanced ductility and improved aesthetic quality of the films through reduction of the characteristic dark color of lignin. Migration tests and disintegration assays of the nanocomposites in simulated composting conditions indicated that, irrespectively of their formulation, the multifunctional nanocomposite films prepared behaved similarly to neat PLA.

## 1. Introduction

In the last years, society concerns on the environmental impact of human activity together with regulations on sustainable development, recyclable and biodegradable materials have prompted more responsibilities and activities involved in the protection of the environment. In this context, the utilization of biodegradable plastics instead of their traditional non-biodegradable counterparts is recognized as one of the ultimate available opportunities to mitigate the environmental problems caused by the disposal of biostable plastic wastes [1].

Among commercially available biodegradable polymers, polylactic acid (PLA) is a linear aliphatic thermoplastic polyester completely derived from renewable agricultural products. PLA is recognized for a close-to-cero carbon footprint and for its ability to be stress and thermally crystallized, impact modified, filled, copolymerized and processed in most conventional polymer processing equipment to produce transparent films, fibers and bottle preforms [2]. The mentioned properties of PLA, as well as its excellent organoleptic characteristics and its suitability for being easily processed by different methods such as injection molding, thermoforming or extrusion, has triggered the use of PLA for food contact and related packaging applications [3,4,5,6]. However, specifically for food packaging applications, PLA-based materials properties may be significantly improved, e.g., in terms of brittleness, crystallization rate, antibacterial and antioxidant activity, UV light blocking, etc. [7]. In this context, in the last years, much effort has been devoted to produce biodegradable functional PLA composites and nanocomposites for packaging applications [6,8,9,10,11].

Among renewable resources that have shown very good performance as PLA filler, lignin, the second most abundant natural polymer on Earth, is recognized for its capability of improving PLA’s thermal stability and mechanical properties, confer antibacterial, antioxidant and UV capacities, and also decrease its water sorption capacity [12,13,14,15,16]. Lignin is an aromatic polymer found in every vascular plant on Earth, constituting 20–30% of lignocellulosic materials weight [17]. Besides its renewable origin, high abundance, biodegradable character and high availability as agroindustrial byproduct [18]; the plentiful functional groups and highly branched polyphenolic aromatic structure of lignin allows it chemical modification and polarity adjustment in such a way that lignin derivatives can be employed in copolymers, blends and composites for a variety of applications such as innovative phenolic resins, epoxies, adhesives and in packaging industry, among others [19,20,21].

Lignin derivatization is recognized as a valuable path to improve its thermo-mechanical properties and achieve specialty uses [20,22]. Particularly, the presence of aromatic and aliphatic hydroxyl groups in lignin facilitates chemical reactions such as esterification, etherification and urethanization [23,24]. Partial or complete derivatization of lignin through its hydroxyl groups is recognized for its usefulness for enhancing lignin compatibility with non-polar polymer matrices [25]. In particular, chemical modification of lignin by etherification and esterification has recently been shown to considerably improve its compatibility with host polyolefins and aliphatic polyesters, enhancing interfacial adhesion and reducing self-association of lignin particles, which in turn resulted in improved mechanical properties of the produced composites [26,27,28].

Particularly for PLA based blends, acetylation of lignin has proved successful for enhancing compatibility of the produced composites, which was attributed to reduced hydrogen-bond strength among lignin molecules upon acetylation [29,30]. PLA/acetylated lignin composites prepared by casting showed more uniform filler size distribution, substantially decreased size of lignin aggregates, much more balanced transparency, UV protection and enhanced mechanical properties [30].

On the other hand, nanofillers are recognized for their ability to improve the stiffness, strength, toughness, thermal stability and/or barrier properties of polymers, even when very low percentages (1–5 wt %) are used. Nanocomposites often perform better than traditional composites because of the higher specific surface area of the fillers [31]. In the last few years, and in view of their potential for improving the physical and mechanical properties of polymer composites, many research efforts have been made to synthesize lignin-based nanoparticles precursors for various nanomaterials manufacturing [32,33,34,35,36,37,38,39,40,41]. Lignin nanoparticles (LNPs) have shown higher antioxidant property, thermal stability and antibacterial property than their parent materials [42]. 

In reference to the use of LNP in biodegradable polyester matrices, they have been employed in PBAT, PLA and glycidyl methacrylate-*g*-PLA based nanocomposites [7,9,43,44,45]. The incorporation of LNP into PLA films processed by both solvent casting and melt extrusion has been attempted. At filler contents of 1 wt % uniform dispersion of LNP in PLA was achieved. However, when the loading content of LNP was set at 3 wt %, aggregation of LNP took place, which was attributed to relatively weak interactions between LNPs and PLA [46].

Overall, and based on the opportunities reviewed related to the incorporation of lignin particles/nanoparticles in polymeric matrices, and the benefits derived from enhanced compatibility between lignin and hydrophobic polymers resulting from esterification and etherification; in the current contribution unmodified (LNP) and chemically modified (i.e., acetylated (aLNP) or treated with citric acid (caLNP)) lignin nanoparticles were used as a nanofiller of PLA films prepared by extrusion. The effect of the nanoparticles on the morphology, tensile and thermal properties of the films was evaluated. Functional properties of importance for food packaging uses such as UV-blocking ability, overall migration, antioxidant and antimicrobial properties were also measured. Finally, the disintegrability of the films in composting conditions was also tested to determine their post-use opportunities.

## 2. Results and Discussion

### 2.1. Lignin Nanoparticles Characterization

**Figure 1:**
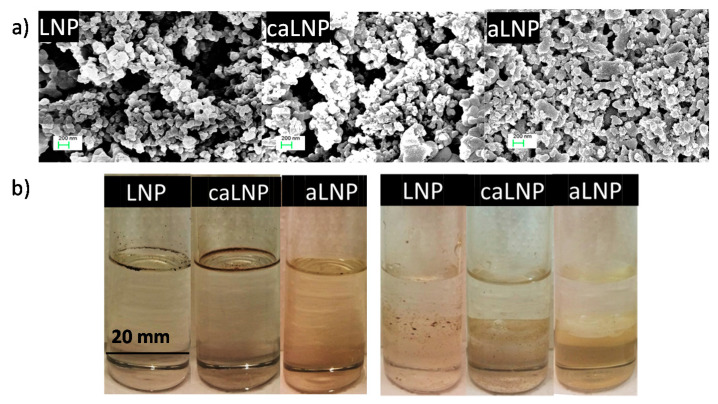
(**a**) FE-SEM micrographs of lignin nanoparticle (LNP), treated with citric acid lignin nanoparticle (caLNP) and acetylated lignin nanoparticle (aLNP) and (**b**) changes in LNP’s polarity resulting from chemical modification: (**left**) LNP, caLNP and aLNP (5 mg) in chloroform (10 mL); (**right**) LNP, caLNP and aLNP (5 mg) in 50/50% *v*/*v* water (upper phase)/chloroform (lower phase) system.

Representative micrographs of pristine and chemically modified lignin nanoparticles are shown in Figure 1a. As it is shown, LNPs appeared as sphere-like particles with characteristic dimensions below 200 nm. In the case of modified LNP, most of them retained the dimensions of the original nanoparticles, while some aggregated elements in the range of 200 and 400 nm were also observed for both aLNP and caLNP.

Chemical modification of LNP was performed according to previous reports [47,48]. In the case of caLNP, simultaneous esterification and etherification has been reported to take place as inferred from FTIR (i.e., new carbonyl peak of ester groups centered at 1745 cm^−1^, ether band centered at 1097 cm^−1^, and concomitant reduction of the absorbance associated with OH groups at 3400–3500 cm^−1^) and from CP/MAS ^13^C NMR spectra (i.e., new resonance at 171 ppm assigned to the carbonyl carbon of ester groups and 89 ppm associated ether structures) [48,49].

Regarding acetylation of LNP, chemical modification was performed according to Eckert et al. who acetylated micrometric kraft softwood lignin [47]. The introduction of acetyl groups in LNP was herein confirmed by FTIR (i.e., new carbonyl peak of ester groups centered at 1750 cm^−1^ and concomitant reduction of the absorbance associated with OH groups at 3400–3500 cm^−1^) and CP/MAS ^13^C NMR spectra (i.e., new resonance at 171 ppm assigned to the carbonyl carbon of esters and a new resonance at 21 ppm attributed to the CH_3_ of acetyl groups) [49,50].

Chemical modification of lignin nanoparticles had a significant effect on their polarity, as inferred from their enhanced compatibility with a typical non-polar organic solvent such as chloroform (Figure 1b). The previous was especially evident for the acetylated lignin nanoparticles, as it is concluded from the homogenous system formed when aLNP were contacted with the organic solvent. The latter became also evident from aLNP’s preference for the organic phase when contacted with an immiscible 50/50% *v*/*v* water/chloroform system. Enhanced compatibility of lignin with chloroform as a consequence of hydrophilic hydroxyl groups replacement with less polar acetyl groups has been previously related to reduced hydrogen-bond strength and aggregation when mixed with PLA [30]. In reference to LNP modified with citric acid, although they showed lower affinity for chloroform than aLNP, compatibility was enhanced with respect to the pristine LNP. The previous was especially evidenced from caLNP’s preference for the organic phase when contacted with the biphasic water/chloroform liquid system. Results thus encouraged the use of modified LNP as fillers of non-polar polymeric matrices such as PLA.

### 2.2. Nanocomposites Characterization

LNP, caLNP and aLNP were used as fillers of PLA at 1 wt % and 3 wt % (Table 1). A hybrid composite containing 0.5 wt % of each modified LNP was also prepared.

#### 2.2.1. Morphological Analysis and Mechanical Properties

**Figure 2:**
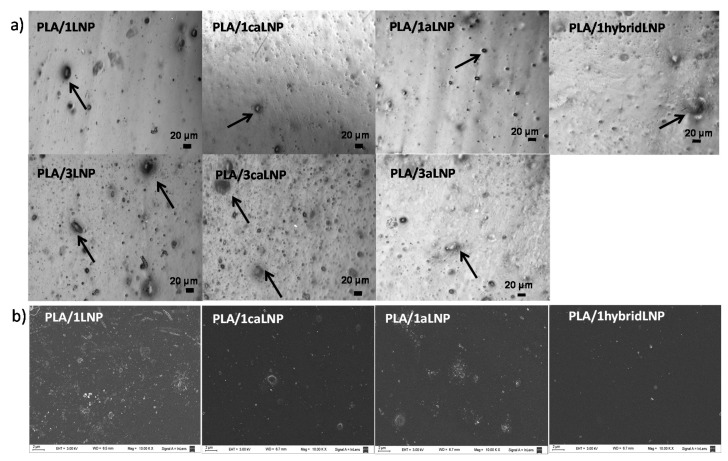
(**a**) Optical micrographs of PLA nanocomposite films and (**b**) FE-SEM micrographs of PLA nanocomposite films with 1 wt % of LNP, caLNP and aLNP.

Optical micrographs of the nanocomposite films prepared as detailed in Table 1 are shown in Figure 2a. As it is shown, in the nanocomposite film with 1 wt % of pristine LNP, nanoparticles are present in the form of aggregates of different sizes. The propensity of natural lignin to aggregate in polymer composites has been attributed to the π-π stacking of its aromatic rings, van der Waals attraction forces of polymer chains and hydrogen bonding among hydroxyl groups [30,51,52,53]. Particularly with regards to composites involving micrometric lignin and PLA, aggregates and impairment of mechanical properties have been attributed to poor compatibility between lignin and PLA [30]. In this framework, modification of micrometric lignin by esterification and etherification has been used to enhance its dispersion in both thermoplastic and thermosetting materials, leading to improved performance of the resulting composites [53,54,55,56].

As it is shown in Figure 2a, when dealing with nanocomposites containing 1 wt % of filler, chemical modification of LNP resulted in better dispersion and reduced aggregates size. The size of lignin aggregates in the PLA matrix has been previously reported to depend on the compatibility between lignin and PLA; the former decreasing when compatibility between phases is improved [30]. Enhanced compatibility of aLNP and caLNP with PLA was expected from the results of the qualitative wettability assays in chloroform, in which both acetylation and modification of LNP with citric acid showed to improve their affinity for non-polar organic phases (Section 2.1). Compatibilization of lignin with PLA through acetylation has been previously reported for micrometric lignin particles [12,29,30,57]. Similar behaviors have also been described for other commercial thermoplastic polymeric matrices such as cellulose triacetate and poly(methyl methacrylate), being also attributed to an improved interaction between components achieved from the acetylation of lignin [31,58].

However, the above-mentioned behavior was no longer evident at 3 wt % of filler, where particle self-aggregation was more pronounced and roughly independent of lignin modification (see arrows in Figure 2a). This could be explained in terms of the recognized higher tendency of nanoparticles to increase their aggregation at higher filler loadings [59,60,61].

Optical microscopy observations were also confirmed by FE-SEM analysis (Figure 2b). Higher particle aggregation was observed for the composite with 1 wt % of unmodified lignin when compared with composites containing aLNP and caLNP. Moreover, in the composites with 3 wt % of filler no clear effect of lignin chemical modification was observed (images not shown).

**Figure 3:**
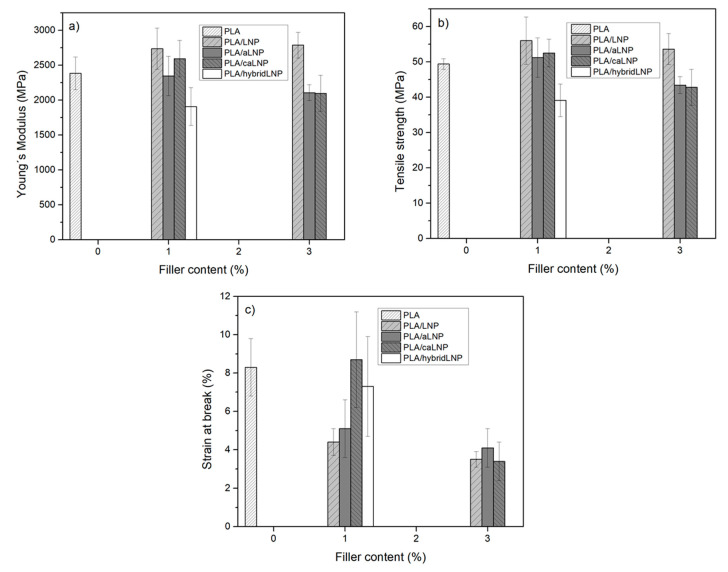
Tensile properties of PLA and PLA nanocomposite films: (**a**) Young’s modulus; (**b**) tensile strength and (**c**) strain at break.

Ductility results were in agreement with microscopic observations (Figure 3). While films with 1 wt % of modified lignin exhibited strain at break values comparable to that of the matrix (especially PLA/1caLNP and PLA/1hybridLNP), the film with unmodified lignin displayed lower ductility as a result of the higher filler aggregation already described, which induced premature failure. The detrimental effect of the incorporation of unmodified lignin into PLA on the material’s ductility has been previously reported by others [1,12,29,30,53,62]. On the other hand, all films with 3 wt % of filler presented much lower ductility values (which were similar among each other) due to increased aggregation. A decrease in the PLA composite’s ductility as a result of the incorporation of higher lignin contents was also observed by Gordobil et al. [12]. Finally, in terms of nanocomposites stiffness and strength, and irrespectively of the type and content of filler, values were mostly in the same range as those of neat PLA (Figure 3).

#### 2.2.2. Thermal Properties

**Figure 4:**
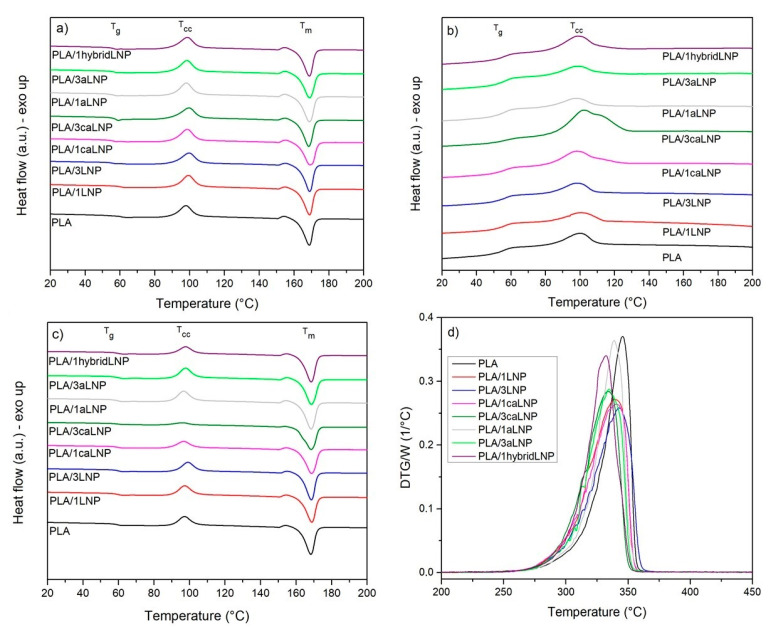
Thermal properties of PLA and PLA nanocomposite films (differential scanning calorimetry (DSC) and derivative thermogravimetric (DTG)): (**a**) first heating; (**b**) cooling; (**c**) second heating and (**d**) DTG data.

The DSC thermograms of neat PLA and the nanocomposite films corresponding to the first heating scan, cooling and second heating scan are shown in Figure 4. Glass transition (T_g_), cold crystallization (T_cc_), melting temperature (T_m_) and crystallinity (X_c_) values are reported in Table 2 along with their deviations. From the first heating scan, it can be observed that neat PLA and PLA composites presented a highly similar thermal behavior, with T_g_ values within the 55–59 °C interval, followed by a small exothermic region located at 98–99 °C, which indicates cold crystallization and, finally, a melting endotherm at 168–169 °C. These values are in agreement with the range of values reported in the literature for both PLA and similar PLA/lignin composites [29,63]. A small exothermic peak (a shoulder crystallization peak) just before the major melting peak was also observed, which can be attributed to the transformation of disordered α´ crystals to the ordered α-form when PLA is crystallized at temperatures corresponding to α´ crystal formation [64]. A similar small recrystallization exotherm preceding the main melting endotherm of PLA has been already reported by several authors [46,65,66]. Different crystalline structures can be developed in PLA, the formation of which depends on the crystallization conditions. The most common α modification develops in conventional melt and solution crystallization conditions. However, only the α´ crystal appears at crystallization temperatures below 100 °C, while crystallization between 100 and 120 °C results in the simultaneous formation of α´ and α crystal structures [67,68,69]. The similarities in the calculated enthalpies of the 1st heating among the films resulted in quite similar crystallinity values of the samples, which were all in the 23–28% interval (Table 1).

During the cooling and second heating scans, no significant differences between the thermal behavior of neat PLA and the nanocomposite films were either observed, except for the nanocomposites containing as filler caLNP, especially at 3 wt %. These materials presented a differential behavior during cooling, where the cold crystallization peak shifted towards higher temperatures values and exhibited a significantly larger area resulting from what appears to be a new high temperature superimposed crystallization peak. The previous, also evident from ∆H_cc_ values in the cooling scan (Table A1), suggests that the particular chemical nature of caLNP favored PLA crystallization during cooling; something that did not take place for LNP and aLNP, which did not induce any evident heterogeneous phase nucleating effect on PLA. During the second heating, no evident cold crystallization peak was observed for the PLA/3caLNP composite, indicating that this film had mostly crystallized during cooling. The effect of lignin particles in the crystallization behavior of PLA is a matter of debate in the literature, with results depending on lignin size (micro/nano), chemical modification processes employed (if any), content and dispersion and composites processing method [29,30,46].

Aiming to determine whether blending with pristine and modified LNP had a deleterious effect on PLA’s thermal stability, thermogravimetric analysis of the nanocomposite films was performed. Figure 4d collects the 1st derivative thermogravimetric (DTG) curves of PLA based nanocomposite films, while the corresponding Tonset and Tmax values are given in Table 2. As it is shown, for all films thermal decomposition took place within the 270–370 °C interval, with minor differences between the corresponding T_onset_ and T_max_ values (i.e., always ≤ 13 °C). These results confirmed that the incorporation of lignin nanoparticles did not have a significant detrimental effect on the thermal stability of PLA.

#### 2.2.3. Optical Properties

**Figure 5:**
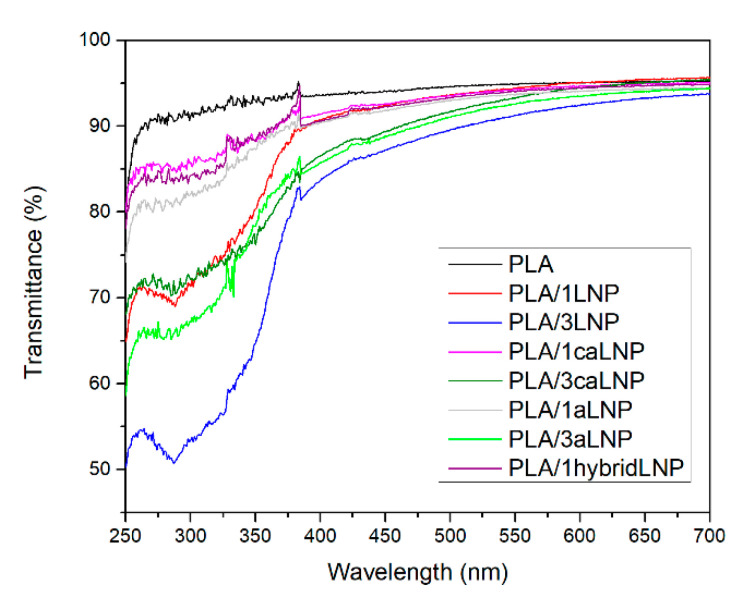
UV–Vis transmittance spectra of PLA and PLA nanocomposites films.

Both ultraviolet and visible light energy may affect certain sensitive food components causing their degradation and inducing changes in the sensory characteristics of the products. In this context, light barrier or interference properties of polymeric materials that are going to be used as food packaging options become of utmost importance [70]. In the case of PLA, it presents high transmittance in both the visible (400–700 nm) and the UV region (250–400 nm) of the spectrum, the previous triggering the study of different strategies for improving its blocking ability [71,72,73,74]. The UV–visible transmittance spectra of neat PLA and the PLA based nanocomposites prepared are shown in Figure 5. All the nanocomposite films showed reduced transmittance compared to neat PLA films, especially in the UV light region (Figure 5). Both nanometric and micrometric lignin particles have been previously recognized for the blocking effect in the UV light region induced when used as fillers of PLA [9,16,43,74]. UV light is subdivided into three distinct wavelength regions: UV-A (400–315 nm), which accounts for the longest wavelength and lowest energy, UV-B (315–280 nm) being the most energetic component of natural UV light and which may cause photochemical degradation of plastics, and UV-C (280–100 nm), which is generally created from artificial light sources [75]. Table 3 summarizes the transmittance of the films at 320 nm, which was herein used as a representative parameter of this property.

In reference to the effect of the nanofiller content, and as expected, all nanocomposites with 3 wt % of filler evidenced a significantly higher UV-blocking effect than those with 1 wt %. Results also showed that the chemical modifications introduced in caLNP and aLNP reduced the UV radiation blocking ability of the resulting composite films (Table 3). Pristine lignin is known as a natural broad-spectrum UV blocker due to its functional groups such as phenolic OH, ketones and other chromophores [76,77,78]. Particularly, the phenolic hydroxyls of lignin are electron-donating groups, which can strengthen the conjugation of benzene rings and provide lignin high UV absorbance [79]. The chemical modifications introduced in LNP involved such chromophores, resulting in a ≈ 30–40% reduction in the UV light absorption of aLNP and caLNP-containing films with respect to PLA/LNP composites. Finally, results evidenced that, irrespective of the type of lignin nanoparticle used as filler, their dispersion within the matrix was good enough to allow the described UV-blocking effect of lignin; differing from previous contributions in which unmodified lignin particles did not significantly reduce the UV light transmission of PLA films as a consequence of their poor dispersion within the matrix [53]. In this sense, lignin dispersion and compatibilization with matrix materials are considered key issues for the development of novel lignin-based UV-protection composites [30].

Changes resulting from the incorporation of pristine and chemically modified lignin nanoparticles into PLA were also evident to the naked eye. While PLA films were transparent and colorless, the nanocomposites containing the unmodified LNP were opaque and dark brown due to their absorption in the blue to violet region of the visible spectrum [43], and the films containing aLNP and caLNP were more transparent and light brownish. The previous is in agreement with other contributions in which PLA films containing pristine lignin were opaque and dark brown, while the PLA/acetylated lignin films were nearly transparent and bright yellow [29,30]. Lignin acetylation has previously proved to produce color and UV absorbance reduction due to the restriction of the electron-donating character of its phenolic hydroxyls [79]. In fact, modification of lignin through acetylation is one of the strategies proposed to minimize the lignin color issue, which is a challenge for its application in food packaging materials where their aesthetic quality influences the consumer acceptability of food products [74]. In this context, a noticeable reduction of the dark brown color of the nanocomposites produced was herein achieved through LNP modification at the expense of an acceptable UV absorption blocking reduction capacity of the films.

#### 2.2.4. Determination of DPPH Radical Scavenging Activity

**Figure 6:**
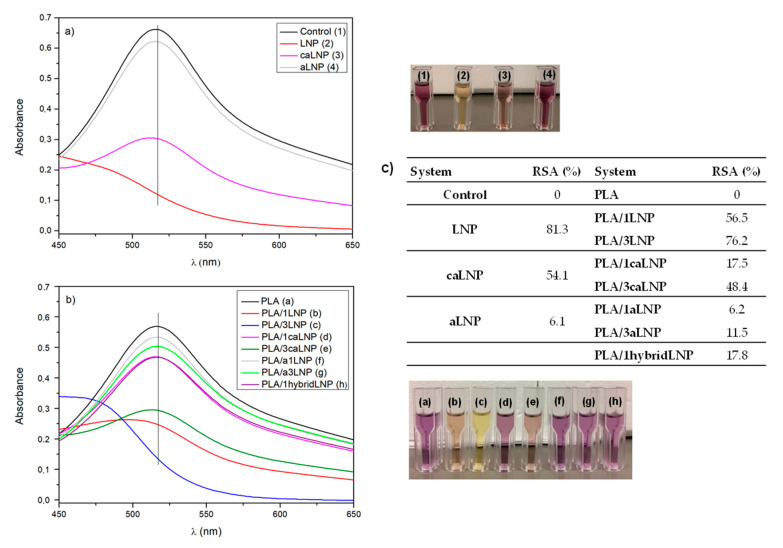
Antioxidant activity evaluated by absorbance of DPPH at 517 nm: (**a**) lignin nanoparticles; (**b**) PLA and PLA nanocomposite films and (**c**) adical scavenging activity (RSA) values of PLA and PLA nanocomposite films.

The antioxidant activity of the nanoparticles and that of PLA based nanocomposite films was measured by evaluation of their DPPH radical scavenging activity (Figure 6). Unmodified LNP were able to trap DPPH radicals, as confirmed by the color change of the DPPH solution from deep violet to pale yellow, indicating the scavenging ability. Research into lignin model compounds indicates that free phenolic hydroxyl groups are essential for its antioxidant activity [80]. In the case of caLNP and aLNP, and in agreement with the changes in their UV-blocking capacities already described, chemical modification involving their phenolic hydroxyl groups reduced their antioxidant property (Figure 6a). The radical scavenging activity of the nanocomposites followed the same trend shown by the nanoparticles (Figure 6b). The PLA films with 1 wt % LNP and 3 wt % LNP exhibited the highest DPPH radical scavenging activity, whereas the nanocomposites containing chemically modified nanoparticles showed reduced antioxidant activity. In addition, for all films, the scavenging activity increased with the nanofiller content.

#### 2.2.5. Overall Migration

While polymeric packaging materials protect food during storage and transportation, the polymeric matrix itself and the chemical compounds incorporated to improve its functionality may interact with food components and migrate into the products during their transport, commercialization and/or shelf-life [81,82,83]. In this framework, of particular concern are nanocomposite films used as food packaging materials because the consumer might be exposed to nanoparticles [82]. Migration tests are a valid analysis to determine the maximum mass range/value that can migrate into food products once in contact with the polymeric material. Migration tests performed herein involved two food simulants, i.e., ethanol at 10% (*v*/*v*) and 50% (*v*/*v*) (simulant A and D1, according to the EU 10/2011, respectively), which were used to determine the behavior of PLA and its composites when in contact with hydrophilic (simulant A) and lipophilic (simulant D1) foodstuffs. Table 4 summarizes the results obtained for the different systems.

Results demonstrated that, irrespective of the film formulation, all values were significantly lower than the migration limits allowed for food contact materials (60 mg kg^−1^ simulant), thus suggesting their applicability for direct contact with food. Moreover, results highlighted that there was no significant increase in migration values resulting from the incorporation of the nanoparticles into the PLA films. In fact, in the case of caLNP nanocomposites, migration values in simulant A were lower than those measured for the neat PLA films.

#### 2.2.6. Disintegration in Compost

Currently, triggered by society concerns and regulations on plastics disposal, one of the most important properties for the packaging sector is the suitability of materials to be disintegrated in compost [84]. In this context, the post-use performance of PLA and PLA nanocomposite films was studied by assaying their disintegration under simulated composting conditions according to the ISO 20200 standard [85].

**Figure 7:**
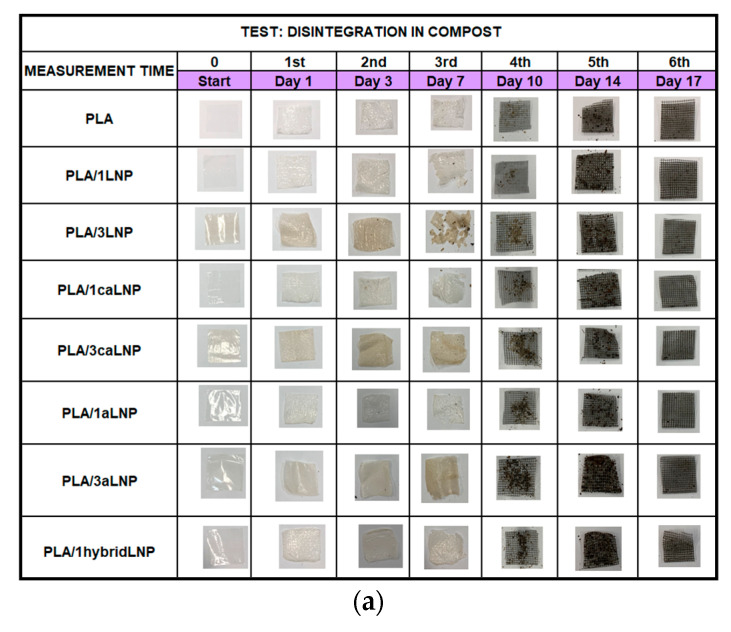

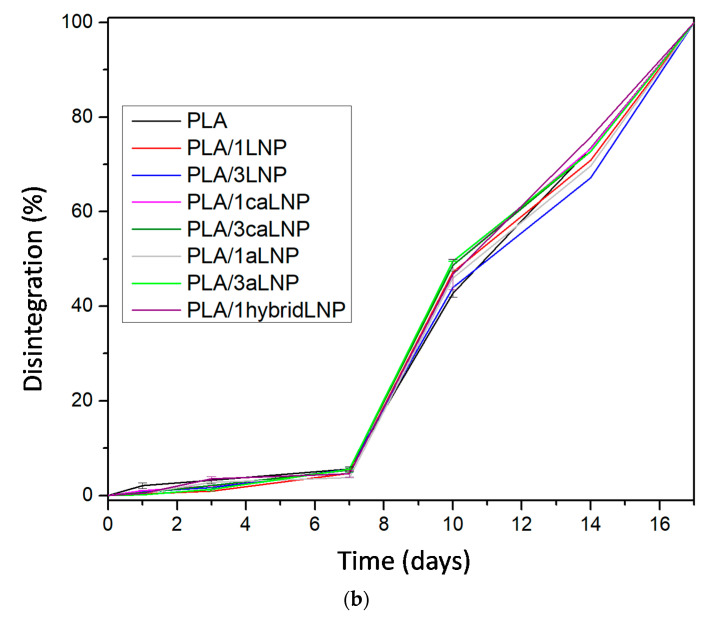
Disintegration of PLA and PLA nanocomposite films in simulated composting conditions (58 °C): (**a**) visual observation and (**b**) disintegration values (wt %).

Figure 7 illustrates the evolution of the films disintegration in compost conditions. Figure 7a shows photographs of all films produced at the initial time of the test and after different incubation periods within 17 days. Aesthetic/optical variations in the films were observed after the first day of incubation. Specifically, deformed and whitish sample surfaces were observed and these effects were more visible after 3 days in composting conditions. The surface whitening of PLA has frequently been read as a signal of hydrolytic degradation, which induces a change in the refractive index of the material as a consequence of water absorption and/or formation of low molecular weight degradation products [86]. Visible fractures appeared at 7 days as previously reported for similar systems [14,87]. Fragmentation continued for all samples assayed during the following days, and after 17 days no considerable amount of remaining materials was visible to the naked eye. Visual observations are in accordance with the disintegration curve depicted in Figure 7b, where values higher than 90% achieved at 17 days of incubation are shown. Moreover, data also showed that the addition of LNP, caLNP and aLNP did not impose significant changes in films disintegration evolution.

#### 2.2.7. Antibacterial Properties

**Figure 8:**
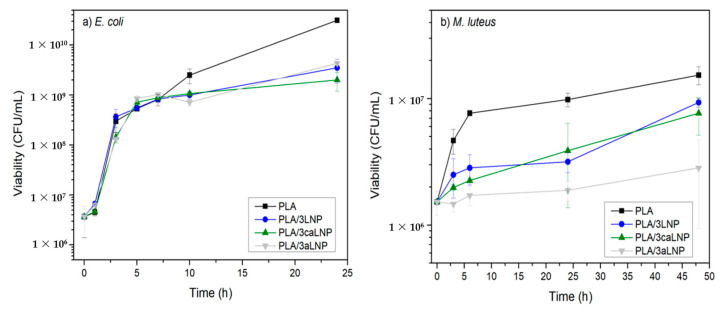
Antibacterial activity tests for PLA and PLA nanocomposite films containing 3 wt % of fillers: (**a**) against *Escherichia coli* and (**b**) against *Micrococcus luteus*.

The antibacterial activity resulting from the addition of pristine and chemically modified LNP into PLA was assayed against Gram-negative and Gram-positive bacteria. With that purpose, the growth of *Escherichia coli* and *Micrococcus luteus* in contact with the different films was evaluated. Results are shown in Figure 8. In general, and irrespective of whether LNP had been chemically modified or not, films containing LNPs induced a decrease in the growth of microorganisms when compared to neat PLA. In the case of *E. coli*, the differences were evident at 10 h, when the broths with LNPs reached the stationary phase of growth while bacteria contacted with the neat PLA film continued growing during the following 14 h. With respect to *M. luteus*, the effect of LNPs mainly influenced the slope of the exponential growth phase, which was significantly smaller for the films containing the nanoparticles. Micrometric lignin has previously proved to be effective against both Gram-positive and Gram-negative bacteria and also against fungi (e.g., *E. coli*, *Saccharomyces cerevisiae*, *Bacillus licheniformis* and *Aspergillus niger,* among others [88,89]). Moreover, and in accordance with the present contribution, Yang et al. showed a significant reduction on the growth of the Gram-negative *Pseudomonas syringae* in a similar system when contacted with PLA/LNP 3 wt % films [9].

Mechanisms through which lignin inhibits microorganisms growth involve enzyme inhibition and cell wall damage caused by polyphenols, concomitantly leading to cellular lysis [9,90]. The latter is related to the lignin type (source/extraction method), and specifically to the presence of phenolic compounds and different functional groups containing oxygen in its structure [9,14,91,92]. The presence of a double bond in α, β positions of the side chain and methyl groups (-C-CH3) in γ position makes the phenolic fragments of lignin an effective antimicrobial agent against microorganisms [14,42]. Moreover, the small size of LNPs may be beneficial to the antimicrobial behavior when compared with the pristine non-nanometric lignin [42]. Due to their small size, lignin nanoparticles may penetrate inside the bacterial cell eluding the cell membrane and, during this process, some monophenolic compounds such as cinnamaldehyde derived from lignin may decrease intracellular pH leading to cell death [42,93].

## 3. Materials and Methods

### 3.1. Materials

Alkali lignin, hydrochloric acid (HCl, 35%), ethylene glycol (CH_2_OH)_2_, citric acid (C_6_H_8_O_7_), methanol (CH_3_OH), 2,2-diphenyl-1-picrylhydrazyl (DPPH) (C_18_H_12_N_5_O_6_) and sodium hypophosphite (NaPO_2_H_2_) were purchased from Merck Life Science (Milano, Italy) while chloroform (CHCl_3_) was purchased from Biopack Productos Quimicos (Ciudad Autónoma de Buenos Aires, Argentina) and acetic anhydride (C_4_H_6_O_3_, 97%) was bought from Cicarelli (San Lorenzo, Argentina). Poly (lactic acid) (PLA 3251D), with a specific gravity of 1.24 g/cm^3^, a relative viscosity of ca. 2.5, and a melt flow index (MFI) of 35 g/10 min (190 °C, 2.16 kg) was supplied by NatureWorks LLC, Minnetonka, Minnesota, USA. All other reagents used were of analytical grade.

### 3.2. Synthesis and Chemical Modification of Lignin Nanoparticles

LNP were obtained from alkaline lignin by treatment with HCl, as detailed in [48,94]. Briefly, a solution of 4% (*w*/*v*) lignin in ethylene glycol was prepared under stirring at 35 °C during 2 h. Then, HCl (8 mL, 0.25 M) was added to 192 mL of the lignin solution at a rate of 2 drop/min. After 2 h, the product was filtered through filter paper (Whatman 541, hole size 22 μm) and the product was dialyzed for 3 days against deionized water until neutral pH and finally freeze-dried.

The LNP were then modified by two different methodologies. On the one hand, 1.7 g of LNP were acetylated with 43 mL of acetic anhydride under stirring at 80 °C during 2 h, following the procedure reported by Eckert et al. for the acetylation of micrometric softwood lignin [47]. The acetylated nanoparticles (aLNP) were washed successively with ethanol and distilled water until pH 6. On the other hand, LNP were also modified by contacting 2 g of LNP during 1 h with 100 mL of citric acid aqueous solution (5 wt %) using sodium hypophosphite as a catalyst (1 wt %). The suspension was then put in a vacuum oven at 0.6 bar for 2 h, kept for 12 h at room temperature and finally air dried at 60 °C during 48 h. The material (caLNP) was then maintained at 130 °C for 4 h, and finally redispersed in water, centrifuged three times and dialyzed against deionized water [95]. After proper ultrasonic treatment both aqueous suspensions of aLNP and caLNP were freeze-dried [48].

### 3.3. Nanoparticles Characterization

Field emission scanning electron microscopy (FESEM): Lignin nanoparticles were examined using a field emission scanning electron microscope (FESEM, Zeiss Supra 40, Dresden, Germany) at an operating voltage of 5 kV. Single drops of LNP, caLNP and aLNP aqueous suspensions were cast onto a silicon substrate, dried during 24 h and gold sputtered before the analysis.

Solid-state CP/MAS ^13^C nuclear magnetic resonance spectroscopy (CP/MAS ^13^C NMR): Solid state CP/MAS ^13^C NMR analysis of native and chemically modified lignin nanoparticles was performed by using a Bruker Avance III 400 NMR spectrometer (BrukerBioSpin AG, City, Switzerland) equipped with a 4-mm MAS probe. The measurements were done at 298.3 K using the ramp ^13^C CP/MAS pulse sequence (cross-polarization and magic angle spinning) with proton decoupling during acquisition. The contact time during CP was set as 2 ms. The SPINAL64 sequence (small phase incremental alternation with 64 steps) was used for hetero nuclear decoupling during acquisition with a proton field H1H. The spinning rate, contact time during CP and relaxation delay were separately set as 12 kHz, 2 ms and 2.0 s.

Fourier transform infrared spectroscopy (FTIR): Fourier transform infrared spectra of pristine and chemically modified lignin nanoparticles were acquired on a Jasco FTIR 615 spectrometer (Jasco Inc., Easton, MD, USA). Carefully dried samples were mixed with previously dried KBr at a 1:500 ratio and pressed into a disc. Samples were scanned 40 times at a resolution of 4 cm^−1^ in the wavenumber range of 4000–500 cm^−1^.

Wettability test: The surface polarity of native and chemically modified lignin nanoparticles was qualitatively assessed by placing samples (5 ± 0.5 mg) into transparent test tubes containing (a) 5 mL of chloroform and (b) equal volumes (5 mL) of water and chloroform. Tubes were shaken and visually inspected to assess the distribution of the samples (upper phase: water, δ = 0.998 g/mL, PI = 10.2; lower phase: chloroform, δ = 1.49 g/mL, polarity index (PI) = 4.1).

### 3.4. Nanocomposites Preparation

Neat PLA pellets and lignin nanoparticles (LNP, caLNP, aLNP; at 1 wt %, 3 wt % and a hybrid with 0.5 wt % of both caLNP and aLNP, Table 1) were introduced into the microextruder (DSM Xplore 15 Micro Compounder, Xplore Instruments BV, Sittard, The Netherlands) and the following parameters were adopted to process the material: screw speed = 100 rpm, temperature=190 °C and previous mixing time = 2 min.

### 3.5. Nanocomposites Characterization

Morphological analysis: The morphology of PLA and PLA nanocomposite films was studied in a field-emission scanning electron microscope (FESEM) (Zeiss Supra 40, Dresden, Germany) operating at 3 kV. Samples (5 mm × 5 mm) were coated with gold before observation. Optical microscopy analysis was also performed using a Nikon Epiphot 300 Microscope at 20×. The obtained micrographs were analyzed with the Nis Elements Software (Nikon Instruments Europe B.V. 1076 ER Amsterdam, The Netherlands).

Mechanical behavior: The mechanical performance of neat PLA and PLA nanocomposite films was evaluated through uniaxial tensile tests performed on rectangular specimens (100 mm × 10 mm) by following UNI ISO 527 standard recommendations at a crosshead speed of 5 mm/min. A load cell of 500 N and an initial gauge length of 25 mm were used. Stress–strain curves were obtained from these tests and Young’s modulus, tensile strength and strain at break values were determined. At least 5 samples were tested for each material and average values and their deviations were reported.

Differential scanning calorimetry (DSC): Thermal analysis was carried out in a differential scanning calorimeter (DSC-Q200 from TA Instruments, New Castle, Delaware, DE 19720, USA) under nitrogen atmosphere. Samples of 6–10 mg were heated from −25 to 210 °C at a heating rate of 10 °C/ min (1st heating scan), hold 2 min at 210 °C to erase thermal history, cooled to −25 °C at a cooling rate of 10 °C/min (cooling scan) and heated again from −25 to 210 °C at a heating rate of 10 °C/min (2nd heating scan). Two replicates were analyzed for each material. Glass transition temperature (T_g_), melting temperature (T_m_) and cold crystallization temperature (T_cc_) values were determined using the TA Instruments Universal Analysis 2000 software (TA Instruments, New Castle, Delaware, DE 19720, USA)

The degree of crystallinity (X_c_) was calculated using Equation (1):

(1) $$X_{c}=\left[\frac{\Delta H_{m}-\Delta H_{\mathrm{cc}}}{\Delta H_{m0}\;\left(1-m_{f}\right)}\right]\times 100$$

where ΔH is the apparent enthalpy for melting or crystallization, ΔH_m0_ is the melting enthalpy of 100% crystalline PLA, with an average value of 93 J g^−1^ and (1 − m_f_) is the weight percent of PLA in the nanocomposite films. 

Thermogravimetric analysis (TGA): Thermogravimetric analysis of PLA and nanocomposite films was conducted in a thermogravimetric analyzer (Seiko Exstar, Tokyo, Japan).

Samples (8–10 mg) were heated from 30 to 600 °C under nitrogen atmosphere (250 mL min^−1^) at a constant heating rate of 10 °C min^−1^. The mass loss (TG) and derivative mass loss (DTG) curves were calculated, and the onset degradation temperature (T_onset_) and the maximum thermal degradation temperature (T_max_) were determined for all samples. The T_onset_ was taken as the temperature at which the sample had lost 5 wt % of its initial mass. The T_max_ was also collected from DTG peaks maxima.

Optical properties: UV and visible light transmittance of the nanocomposites were measured using a Varian (Cary 4000, SpectraLab Scientific Inc., Markham, ON, Canada) ultraviolet–visible (UV–Vis) spectrophotometer in the 250–700 nm interval at room temperature, employing a scan speed of 240 nm/min. Transmittance curves were obtained for each material and the correspondent values at 320 nm were employed to determine UV light barrier properties.

DPPH radical scavenging activity: Small pieces of the films (0.1 g) were immersed in 2 mL of methanol at room temperature for 24 h, after which the supernatant was collected for evaluation of DPPH radical scavenging activity. An aliquot of 1 mL of the supernatant was mixed with DPPH in methanol (1 mL, 50 mg/L). The absorbance of DPPH over time was measured at 517 nm in a UV–vis spectrometer (Varian, Cary 4000, SpectraLab Scientific Inc., Markham, ON, Canada) ultraviolet–visible (UV–Vis) using proper controls. The DPPH radical scavenging activity (RSA) of the nanocomposite films was calculated according to Equation (2):

(2) $$\mathrm{Antiradical}\;\mathrm{activity}\;\left(\mathrm{RSA},\%\right)=\left(1-\frac{A_{\mathrm{sample}}}{A_{\mathrm{control}}}\right)\times 100$$

Overall migration: Overall migration tests of the films (10 cm^2^) were done in triplicate in 10 mL of food simulant A (10% (*v*/*v*) ethanol/water solution) and food simulant D1 (50% (*v*/*v*) ethanol/water solution). Samples were kept in the ethanol solutions in a controlled atmosphere at 40 °C during 10 days, according to European Commission Regulation EU 10/2011. At last, films were removed and the simulants evaporated. The residues were weighed in an analytical balance with ±0.01 mg accuracy and the migration value in mg/kg of each simulant was calculated.

Disintegrability in composting conditions: The disintegrability of PLA and PLA nanocomposite films in simulated composting conditions was evaluated according to the ISO-20200 standard. A specific quantity of compost supplied by Gesenu S.P.A. (Gesenu SpA, Perugia, Italy) was mixed with the synthetic biowaste prepared with sawdust, rabbit food, starch, sugar, oil and urea. The water content of the substrate was around 50 wt % and the aerobic conditions were guaranteed by mixing it gently. The samples (25 mm × 25 mm) were buried at 4–6 cm depth in perforated boxes containing the prepared mix and incubated at 58 °C during 17 days. The samples were recovered at different intervals, washed with distilled water, dried in an oven at 37 °C for 24 h, and weighed. The disintegrability values (wt %) at different periods of incubation were obtained by normalizing the sample weight to the initial weight.

Antibacterial activity: The antibacterial activity of PLA and nanocomposites films was evaluated at the highest content of filler (PLA/3LNP, PLA/3caLNP and PLA/3aLNP). In order to assay the effectiveness on both Gram positive and Gram negative bacteria, each nanocomposite film was tested against *Micrococcus luteus* NCTC 196 and *Escherichia coli* ATCC 8739, respectively. The PLA neat film was assayed as the control. Film samples (rectangles of 3.24 cm^2^) were irradiated with UV light and tested in tubes containing 5 mL of inoculated medium (Meat Broth) with 10^6^ cells/mL. All tubes were placed on a reciprocal shaker at 100 rpm and incubated for 24 h at 37 ± 0.5 °C for *E. coli* cultures, and at 28 ± 1 °C for 48 h for *M. luteus*. Culture conditions and sampling time were selected according to the optimum growth temperature and growth rate of each microorganism. Serial dilutions of the samples were plated in triplicate on meat agar and CFU (colony forming unit) counts after incubation at the corresponding temperatures for 24 h and 48 h were recorded.

## 4. Conclusions

In the current contribution, lignin nanoparticles (pristine and chemically modified) were used in the preparation of PLA composites processed by extrusion. Chemical modification through acetylation and by treatment of LNP with citric acid had a significant effect on nanoparticles’ polarity as inferred from their enhanced compatibility with chloroform and, at 1 wt %, their better dispersion and reduced aggregates size in PLA, concomitantly with the enhanced ductility of the nanocomposites when compared with the films containing pristine LNP. In terms of thermal behavior, caLNP showed to favor PLA crystallization during cooling, whereas characteristic decomposition temperatures indicated that the incorporation of lignin nanoparticles did not have a significant detrimental effect on the thermal stability of PLA.

On the other hand, the addition of lignin nanoparticles conferred UV-blocking and antioxidant and antibacterial properties to the films, especially at the higher filler loading assayed. Comparison of results among the different nanoparticles used showed that the chemical modifications introduced in caLNP and aLNP partially reduced the UV radiation blocking ability and the antioxidant property of the resulting composites, whereas the characteristic dark color of the films containing pristine LNP could be noticeably reduced by chemical modification. Finally, migration tests and disintegration assays in simulated composting conditions indicated that, irrespective of the film formulation, the behavior of the nanocomposite films was similar to that of PLA, with migration values significantly lower than the migration limits allowed for food contact materials and disintegration levels at 17 days above 90%. Overall, the addition of lignin nanoparticles (pristine or chemically modified) to PLA showed to be an interesting alternative to produce promising multifunctional PLA based nanocomposites for food packaging applications.

## Figures and Tables

**Table 1:** PLA nanocomposites composition.

Formulation	PLA	LNP	caLNP	aLNP
	wt %	wt %	wt %	wt %
PLA	100	0	0	0
PLA/1LNP	99	1	0	0
PLA/3LNP	97	3	0	0
PLA/1caLNP	99	0	1	0
PLA/3caLNP	97	0	3	0
PLA/1aLNP	99	0	0	1
PLA/3aLNP	97	0	0	3
PLA/1hybridLNP	99	0	0.5	0.5

**Table 2:** T_onset_ and T_max_ values calculated for PLA and PLA nanocomposite films.

Formulation	T_onset_ (°C)	T_max_ (°C)
PLA	293	345
PLA/1LNP	290	339
PLA/3LNP	291	342
PLA/1caLNP	289	341
PLA/3caLNP	286	334
PLA/1aLNP	295	339
PLA/3aLNP	293	335
PLA/1hybridLNP	292	332

**Table 3:** Transmittance (%) at 320 nm of PLA and PLA nanocomposite films.

Formulation	**Transmittance** (%)
PLA	93
PLA/1LNP	75
PLA/3LNP	56
PLA/1caLNP	86
PLA/3caLNP	74
PLA/1aLNP	84
PLA/3aLNP	70
PLA/1hybridLNP	85

**Table 4:** Overall migration values in Simulants A and D1 (10% (*v*/*v*) and 50% (*v*/*v*) ethanol, respectively) for PLA and PLA nanocomposite films.

Formulations	Overall Migration	
	Ethanol 10% (*v*/*v*) (mg kg^−1^) @ 10 days 40 °C	Ethanol 50% (*v*/*v*) (mg kg^−1^) @ 10 days 40 °C
PLA	13.3 ± 2.3	13.4 ± 0.3
PLA/1LNP	13.1 ± 1.4	12.5 ± 2.2
PLA/3LNP	13.9 ± 0.9	15.4 ± 1.3
PLA/1caLNP	9.4 ± 0.9	13.4 ± 0.9
PLA/3caLNP	9.6 ± 0.6	13.2 ± 1.3
PLA/1aLNP	13.7 ± 0.6	13.4 ± 0.9
PLA/3aLNP	12.1 ± 1.7	11.9 ± 1.3
PLA/1hybridLNP	13.1 ± 2.0	12.5 ± 0.9

## Data Availability

The data presented in this study are available on request from the corresponding author

## Sample Availability

Samples of the compounds are available from the authors.

## Appendix A

**Table A1:** Thermal parameters from DSC analysis of PLA and PLA nanocomposite films.

	1st Heating Scan						Cooling	
Formulation	T_g_ (°C)	T_cc_ (°C)	∆Hcc (J/g)	T_m_ (°C)	∆H_m_ (J/g)	X_c_ (%)	T_cc_ (°C)	∆H_cc_ (J/g)
PLA	58.9 ± 2.0	98.1 ± 0.3	27.1 ± 0.3	168.9 ± 0.1	48.7 ±0.1	23.0 ± 0.2	98.7 ±1.4	7.2 ± 1.4
PLA/1LNP	58.9 ± 2.0	99.3 ±0.1	25.0 ± 0.1	168.8 ±0.1	46.8 ± 0.7	23.3 ± 0.7	99.1 ± 2.4	6.3 ± 2.7
PLA/3LNP	56.3 ± 1.2	99.3 ± 0.6	26.6 ± 0.1	168.7 ± 0.3	47.8 ± 0.4	22.6 ± 0.4	97.9 ± 0.6	6.9 ± 1.7
PLA/1caLNP	56.4 ± 1.2	99.1 ± 0.7	27.0 ± 0.1	169.4 ± 0.3	48.2 ± 2.1	22.7 ± 2.1	98.3 ± 0.1	13.8 ± 1.5
PLA/3caLNP	56.3 ± 1.3	99.5 ± 0.1	27.8± 0.9	168.6 ± 0.2	50.4 ± 0.4	24.1 ± 0.5	102.1 ± 0.1	28.8 ± 0.3
PLA/1aLNP	55.4 ± 1.4	98.1 ± 0.1	24.3 ± 5.5	168.51 ± 0.4	48.9 ± 0.9	26.3 ± 6.9	96.4 ± 1.7	5.2 ± 2.6
PLA/3aLNP	55.0 ± 2.0	98.3 ± 0.3	27.2 ± 0.3	168.5 ± 0.4	50.2 ± 2.1	24.5 ± 1.9	98.9 ± 0.5	7.8 ± 0.3
PLA/1hybridLNP	58.1 ± 2.7	98.1 ± 0.7	24.2 ± 4.3	169.0 ± 0.4	50.3 ± 0.9	27.9 ± 5.5	98.8 ± 0.5	13.0 ± 0.5
	**2nd Heating Scan**							
	**T_g_ (°C)**	**T_cc_ (°C)**	**∆H_cc_ (J/g)**	**T_m_ (°C)**	**∆H_m_ (J/g)**	**X_c_ (%)**		
PLA	59.4 ± 0.6	97.1 ± 0.1	19.0 ± 1.2	168.4 ± 0. 2	41.3 ± 0.6	30.1 ± 0.6		
PLA/1LNP	59.3 ± 0.2	97.6 ± 0.5	21.3 ±0.3	168.6 ± 0.3	46.4 ± 0.4	26.8 ± 0.8		
PLA/3LNP	59.7 ± 0.2	99.1 ± 0.1	20.7 ±1.5	168.5 ± 0.2	47.4 ± 0.4	28.5 ± 2.1		
PLA/1caLNP	59.5 ± 0.3	97.1 ± 0.4	15.0 ± 0.2	168.6 ± 0.2	47.2 ± 1.3	34.4 ± 1.6		
PLA/3caLNP	59.0 ± 0.1	95.9 ± 0.2	5.2 ± 0.2	168.6 ± 0.1	47.6 ± 0.6	45.3 ± 0.8		
PLA/1aLNP	59.4 ± 0.4	97.3 ± 0.9	21.7 ± 0.7	168.6 ± 0.1	49.7 ± 0.2	29.9 ± 1.0		
PLA/3aLNP	59.5 ± 0.3	97.4 ± 0.5	19.5 ± 0.6	168.5 ± 0.3	40.5 ± 1.9	32.1 ± 2.7		
PLA/1hybridLNP	59.1 ± 0.1	97.8 ± 0.1	14.4 ± 1.2	168.5 ± 0.1	47.8 ± 0.2	35.6 ± 1.5		
